# Redox Metabolism in Ecophysiology and Evolution, 2nd Edition

**DOI:** 10.3390/antiox14060755

**Published:** 2025-06-19

**Authors:** Marko D. Prokić, Marcelo Hermes-Lima, Daniel C. Moreira

**Affiliations:** 1 Department of Physiology, Institute for Biological Research “Siniša Stanković”, National Institute of Republic of Serbia, University of Belgrade, Bulevar Despota Stefana 142, 11060 Belgrade, Serbia; marko.prokic@ibiss.bg.ac.rs; 2 Department of Cell Biology, University of Brasilia, Brasilia 70910-900, Brazil; hermes@unb.br; 3 Research Center in Morphology and Applied Immunology, Faculty of Medicine, University of Brasilia, Brasilia 70910-900, Brazil

The ability of organisms to regulate the production of reactive oxygen and nitrogen species (RONS), manage pro-oxidant activity, and make use of redox pathways has significantly influenced their evolution [1]. Redox signaling is mediated by reactive species, redox-sensitive transcription factors, and redox-sensitive proteins, all forming an interconnected network of signaling pathways [2]. These pathways play crucial roles in various essential processes in aerobic organisms [3]. Furthermore, changes in oxidative status in response to unpredictable or fluctuating environmental conditions have been linked to alterations in life-history traits such as growth, reproduction, and longevity [4]. Therefore, the plasticity of the oxidative stress response may be vital in determining whether organisms survive, adapt, or thrive in daily/seasonally changing environments.

Despite the increasing interest in evolutionary ecology and ecophysiology regarding these responses, significant gaps in understanding remain. The second edition of “Redox Metabolism in Ecophysiology and Evolution” continues to explore the adaptive responses of redox metabolism in organisms facing environmental stressors. This issue provides new insights into the interactions between oxidative stress, organism fitness and ecosystem health. These studies, produced by authors based in five countries (Chile, Brazil, Mexico, New Zealand, and South Korea), are of relevance for understanding of molecular and biochemical mechanisms involved in adaptation to natural or man-altered environments.

Variability in environmental conditions in estuarine ecosystems creates a range of physiological stressors, particularly for sessile organisms. Understanding how these organisms respond to changing environments provides valuable information for the management of estuarine areas. In their study, Cruces and co-workers from Chile (contribution 1) investigated the effects of the tidal cycle, solar radiation, and salinity fluctuations on the photosynthetic and cellular responses of the symbiont complex (*A. hermaphroditica/P. anthopleurum*) over a 24 h period, both in the field—at the Quempillén River Estuary in Chile—and in a laboratory setting. Their findings revealed that the photosynthetic parameters of the symbiont complex decreased with increasing ambient radiation; however, no relationship was observed with changes in salinity. The cellular responses, such as lipid peroxidation and total antioxidant capacity, measured during day and dark periods, were primarily linked to increased levels of ultraviolet radiation (UVR), with a lesser impact from photosynthetically active radiation (PAR). This study highlighted a series of metabolic adjustments that the symbiont complex undergoes at different times throughout the day to minimize the harmful effects of environmental stress, particularly from UVR.

Insect metamorphosis involves significant metabolic and physiological changes. A study by Moreira and Hermes-Lima (contribution 2), from Brazil, addressed several questions regarding the changes in the antioxidant system, redox balance, and the role of endogenous antioxidants during metamorphosis, specifically in the larva-to-pupa and pupa-to-adult transitions of holometabolous insects. Using the sunflower patch butterfly (*Chlosyne lacinia*) as a model, their findings revealed fluctuations in redox balance throughout metamorphosis, characterized by periods of oxidative eustress and antioxidant responses. The transition from larva to pupa includes increased anaerobic capacity, oxidative damage, and the activation of antioxidant enzymes. As metamorphosis progresses, there is a return to a more reduced oxidative state. The adult emergence phase is distinguished by an increase in oxidative metabolism, which is managed by enhanced antioxidant response. Findings underscore the integral role of redox adaptations and oxidative stress management in insect development. This research provides novel insights into the biochemical processes underpinning complete metamorphosis and highlights the importance of redox balance in the process.

In the context of environmental pollution, water temperature and pesticide use are expected to exert synergistic effects, underscoring the need to assess their effects on aquatic organisms. A study by Montory and co-workers (contribution 3), a team from Chile and Mexico, examined the combined effects of the pesticide azamethiphos (concentrations of 15 µg L^−1^ and 100 µg L^−1^), and temperature (12 °C and 15 °C) on the gonads and gills of the oyster *Ostrea chilensis*. The authors measured oxidative damage and total antioxidant capacity during a 7-day treatment period. The results revealed that both the pesticide exposure and the duration of exposure could increase lipid peroxidation in the examined tissues, as well as protein carbonylation in the gonads. In the gills, all treatment conditions resulted in elevated levels of oxidative damage to proteins. Interestingly, temperature did not have significant effects on oxidative damage. However, in the case of antioxidant response, it happened only in the gonads following temperature treatment. Although alterations in oxidative stress can arise from multiple environmental stressors, this study indicates that the duration of exposure to pesticide may have a greater influence on this oyster species than temperature.

The emersion of marine organisms during transport presents a significant physiological challenge, often resulting in increased mortality rates due to prolonged periods of stress from emersion. In their research, Delorme and co-workers (contribution 4), from New Zealand, investigated how different hardening treatments (short, long, and intermittent sub-lethal emersion) affect various traits of juvenile New Zealand green-lipped mussels (*Perna canaliculus*). The authors examined oxidative status through markers such as protein carbonyls, lipid hydroperoxides, 8-hydroxydeoxyguanosine, and antioxidant enzymes. They also assessed resettlement behavior, respiration rates, and survival after transport and during recovery. The results indicated that hardening treatments can produce a range of physiological responses in the mussels. Longer and intermittent sub-lethal emersion resulted in low viability, reduced resettlement, and increased oxidative damage. In contrast, subjects subjected to short emersion showed no significant stress, displayed an enhanced antioxidant response, and exhibited greater resettlement behavior. These individuals remained viable for 24 h following immersion in seawater after transport. The study’s analysis of oxidative stress markers revealed specific emersion durations and recovery periods that can optimize hardening effects while minimizing irreversible oxidative damage. Consequently, this research underscores the importance of monitoring oxidative biomarkers as a valuable tool for refining mussel transportation protocols. Such procedures could ameliorate aquaculture performance.

In their paper, Kim and co-workers (contribution 5), from New Zealand and South Korea, highlighted the roles of reactive oxygen species (ROS) and NADPH oxidases in red algae. They explored the roles of ROS and calcium signaling in fertilization, wound healing, environmental stress responses, and developmental processes. Notably, they discussed the evolutionary divergence of NADPH oxidases in red algae and their specialization in supporting adaptive traits. The understanding of NADPH oxidase at the molecular level is crucial for investigating physiological processes and adaptive strategies of red algae. The findings underscore the importance of ROS signaling and NADPH oxidase activity in red algal physiology, with broader implications for understanding redox-regulated adaptation in aquatic systems.

Together, these studies advance our understanding of redox regulation in diverse organisms. By integrating ecophysiological and molecular approaches, this issue emphasizes the significance of oxidative stress biology in shaping how organisms interact with dynamic environments and stressors. We hope these contributions pave the way for further interdisciplinary work on redox metabolism, evolution, and environmental adaptation.
